# Supplementary material for: HIVprotI: an integrated web based platform for prediction and design of HIV proteins inhibitors
Source: J Cheminform. 2018 Mar 9;10:12. doi: 10.1186/s13321-018-0266-y (PMC5845081; doi:10.1186/s13321-018-0266-y)
Supplement: Supplementary file 2 — Additional file 2. Source code of HIVProtI web server. [file 13321_2018_266_MOESM2_ESM.zip › HIVprotI_Source-code/hivprotI/weka-3-6-6/doc/weka/associations/PriorEstimation.html]

PriorEstimation


---


|  |  |  |
| --- | --- | --- |
| **Overview** \| **Package** \| **Class** \| **Tree** \| **Deprecated** \| **Index** \| **Help** \| **Weka's home** | |  |
| **PREV CLASS**   **NEXT CLASS** | **FRAMES**    **NO FRAMES**     **All Classes** |  |
| SUMMARY: NESTED \| FIELD \| CONSTR \| METHOD | DETAIL: FIELD \| CONSTR \| METHOD |  |


---


## weka.associations Class PriorEstimation

```
java.lang.Object
  weka.associations.PriorEstimation
```

**All Implemented Interfaces:**: java.io.Serializable, RevisionHandler

---

``` public class PriorEstimation extends java.lang.Object implements java.io.Serializable, RevisionHandler ```

Class implementing the prior estimattion of the predictive apriori algorithm
for mining association rules.
Reference: T. Scheffer (2001). *Finding Association Rules That Trade Support
Optimally against Confidence*. Proc of the 5th European Conf.
on Principles and Practice of Knowledge Discovery in Databases (PKDD'01),
pp. 424-435. Freiburg, Germany: Springer-Verlag.

**Version:**
:   $Revision: 1.7 $

**Author:**
:   Stefan Mutter (mutter@cs.waikato.ac.nz)

**See Also:**: Serialized Form

---

**Constructor Summary**

`PriorEstimation(Instances instances,
int numRules,
int numIntervals,
boolean car)`
  
          Constructor


| **Method Summary** | |
| --- | --- |
| `RuleItem` | `addCons(int[] itemArray)`             generates a class association rule out of a given premise. |
| `void` | `buildDistribution(double conf, double length)`             updates the distribution of the confidence values. |
| `double` | `calculatePriorSum(boolean weighted, double mPoint)`             calculates the numerator and the denominator of the prior equation |
| `java.util.Hashtable` | `estimatePrior()`             Method to estimate the prior probabilities |
| `double` | `findIntervall(double conf)`             searches the mid point of the interval a given confidence value falls into |
| `void` | `generateDistribution()`             Calculates the prior distribution. |
| `double[]` | `getMidPoints()`             returns an ordered array of all mid points |
| `java.lang.String` | `getRevision()`             Returns the revision string. |
| `static double` | `logbinomialCoefficient(int upperIndex, int lowerIndex)`             Method that calculates the base 2 logarithm of a binomial coefficient |
| `double` | `midPoint(double size, int number)`             calculates the mid point of an interval |
| `void` | `midPoints()`             split the interval [0,1] into a predefined number of intervals and calculates their mid points |
| `int[]` | `randomCARule(int maxLength, int actualLength, java.util.Random randNum)`             Constructs an item set of certain length randomly. |
| `int[]` | `randomRule(int maxLength, int actualLength, java.util.Random randNum)`             Constructs an item set of certain length randomly. |
| `RuleItem` | `splitItemSet(int premiseLength, int[] itemArray)`             splits an item set into premise and consequence and constructs therefore an association rule. |
| `void` | `updateCounters(ItemSet itemSet)`             updates the support count of an item set |

**Methods inherited from class java.lang.Object**

`equals, getClass, hashCode, notify, notifyAll, toString, wait, wait, wait`

**Constructor Detail**

### PriorEstimation

```
public PriorEstimation(Instances instances,
                       int numRules,
                       int numIntervals,
                       boolean car)
```

:   Constructor

    **Parameters:**: `instances` - the instances to be used for generating the associations: `numRules` - the number of random rules used for generating the prior: `numIntervals` - the number of intervals to discretise [0,1]: `car` - flag indicating whether standard or class association rules are mined


**Method Detail**

### generateDistribution

```
public final void generateDistribution()
                                throws java.lang.Exception
```

:   Calculates the prior distribution.

    :   **Throws:**: `java.lang.Exception` - if prior can't be estimated successfully

---


### randomRule

```
public final int[] randomRule(int maxLength,
                              int actualLength,
                              java.util.Random randNum)
```

:   Constructs an item set of certain length randomly.
    This method is used for standard association rule mining.

    :   **Parameters:**: `maxLength` - the number of attributes of the instances: `actualLength` - the number of attributes that should be present in the item set: `randNum` - the random number generator **Returns:**: a randomly constructed item set in form of an int array

---


### randomCARule

```
public final int[] randomCARule(int maxLength,
                                int actualLength,
                                java.util.Random randNum)
```

:   Constructs an item set of certain length randomly.
    This method is used for class association rule mining.

    :   **Parameters:**: `maxLength` - the number of attributes of the instances: `actualLength` - the number of attributes that should be present in the item set: `randNum` - the random number generator **Returns:**: a randomly constructed item set in form of an int array

---


### buildDistribution

```
public final void buildDistribution(double conf,
                                    double length)
```

:   updates the distribution of the confidence values.
    For every confidence value the interval to which it belongs is searched
    and the confidence is added to the confidence already found in this
    interval.

    :   **Parameters:**: `conf` - the confidence of the randomly created rule: `length` - the legnth of the randomly created rule

---


### findIntervall

```
public final double findIntervall(double conf)
```

:   searches the mid point of the interval a given confidence value falls into

    :   **Parameters:**: `conf` - the confidence of a rule **Returns:**: the mid point of the interval the confidence belongs to

---


### calculatePriorSum

```
public final double calculatePriorSum(boolean weighted,
                                      double mPoint)
```

:   calculates the numerator and the denominator of the prior equation

    :   **Parameters:**: `weighted` - indicates whether the numerator or the denominator is calculated: `mPoint` - the mid Point of an interval **Returns:**: the numerator or denominator of the prior equation

---


### logbinomialCoefficient

```
public static final double logbinomialCoefficient(int upperIndex,
                                                  int lowerIndex)
```

:   Method that calculates the base 2 logarithm of a binomial coefficient

    :   **Parameters:**: `upperIndex` - upper Inedx of the binomial coefficient: `lowerIndex` - lower index of the binomial coefficient **Returns:**: the base 2 logarithm of the binomial coefficient

---


### estimatePrior

```
public final java.util.Hashtable estimatePrior()
                                        throws java.lang.Exception
```

:   Method to estimate the prior probabilities

    :   **Returns:**: a hashtable containing the prior probabilities **Throws:**: `java.lang.Exception` - throws exception if the prior cannot be calculated

---


### midPoints

```
public final void midPoints()
```

:   split the interval [0,1] into a predefined number of intervals and calculates their mid points

---


### midPoint

```
public double midPoint(double size,
                       int number)
```

:   calculates the mid point of an interval

    :   **Parameters:**: `size` - the size of each interval: `number` - the number of the interval. The intervals are numbered from 0 to m\_numIntervals. **Returns:**: the mid point of the interval

---


### getMidPoints

```
public final double[] getMidPoints()
```

:   returns an ordered array of all mid points

    :   **Returns:**: an ordered array of doubles conatining all midpoints

---


### splitItemSet

```
public final RuleItem splitItemSet(int premiseLength,
                                   int[] itemArray)
```

:   splits an item set into premise and consequence and constructs therefore
    an association rule. The length of the premise is given. The attributes
    for premise and consequence are chosen randomly. The result is a RuleItem.

    :   **Parameters:**: `premiseLength` - the length of the premise: `itemArray` - a (randomly generated) item set **Returns:**: a randomly generated association rule stored in a RuleItem

---


### addCons

```
public final RuleItem addCons(int[] itemArray)
```

:   generates a class association rule out of a given premise.
    It randomly chooses a class label as consequence.

    :   **Parameters:**: `itemArray` - the (randomly constructed) premise of the class association rule **Returns:**: a class association rule stored in a RuleItem

---


### updateCounters

```
public final void updateCounters(ItemSet itemSet)
```

:   updates the support count of an item set

    :   **Parameters:**: `itemSet` - the item set

---


### getRevision

```
public java.lang.String getRevision()
```

:   Returns the revision string.

    :   **Specified by:**: `getRevision` in interface `RevisionHandler`
    :   **Returns:**: the revision


---


|  |  |  |
| --- | --- | --- |
| **Overview** \| **Package** \| **Class** \| **Tree** \| **Deprecated** \| **Index** \| **Help** \| **Weka's home** | |  |
| **PREV CLASS**   **NEXT CLASS** | **FRAMES**    **NO FRAMES**     **All Classes** |  |
| SUMMARY: NESTED \| FIELD \| CONSTR \| METHOD | DETAIL: FIELD \| CONSTR \| METHOD |  |


---
